# Synthesis, spectroscopic investigation, theoretical insights *via* DFT and biological assessment of some isatin-based metal complexes

**DOI:** 10.1038/s41598-026-41979-1

**Published:** 2026-04-22

**Authors:** Ohyla A. EL-Gammal, Hanaa A. El-Boraey, Dina A. Tolan

**Affiliations:** 1 Department of Pathology, University Hospital, Menoufia University, Shebin El-Kom, 32511 Egypt; 2 Department of Chemistry, Faculty of Science, Menoufia University, Shebin El-Kom, 32511 Egypt; 3 Department of Chemistry, College of Science and Humanities, Prince Sattam bin Abdulaziz University, Alkharj, 11942 Saudi Arabia

**Keywords:** Complexes, Spectral and thermal, DFT, Antidiabetic, Anticancer, Antibacterial efficacy, Biochemistry, Cancer, Chemistry, Drug discovery

## Abstract

In this work, new VO^2+^, Ni^2+^ and Cu^2+^ complexes (**1–3**), respectively, were synthesized from indoline-2,3-dione with 4,4′-diaminodiphenyl ether. The metal complexes were characterized using elemental analyses (C, H, and N), Fourier transform infrared (FT-IR), UV–Visible spectra, thermogravimetric analysis methods,^1^H-NMR spectrum, molar conductivity and magnetic susceptibility measurements. The findings showed a square pyramidal geometry around the VO^2+^ complex, tetrahedral and distorted square planar environments around the Ni^2+^ Cu^2+^ complexes. Density functional theory (DFT) calculations were performed to elucidate the geometrical and energetic properties of the ligand and its metal complexes. *In vitro* antidiabetic property of the synthesized ligand and its metal chelates (**1–3**) was estimated by α-amylase inhibition method. Investigating the compounds’ *in vitro* anticancer potential against the hepatic cancer cell lines (HepG-2) and against human lung fibroblast normal cell line (WI-38) was reported. The results showed that VO^2+^ complex possesses the highest ability to inhibit α-amylase as well as hepatic cancer cells. In light of this, VO^2+^ complex (**1**) that was synthesized has the potential to be utilized as possible candidates for antidiabetic and hepatic cancer treatment. The *in-vitro* antibacterial efficacy of the synthesized ligand and its metal complexes (**1–3**) was assessed against both two Gram-positive bacteria (*B. subtilis* and *S. aureus*) and two Gram-negative bacteria (*E. coli* and *K. pneumonia*). Cu^2+^ chelate showed the highest activity against the tested microbes.

**Supplementary Information:**

The online version contains supplementary material available at 10.1038/s41598-026-41979-1.

## Introduction

Recent decades have witnessed a growing interest in the use of metal chelates for medicinal purposes. Their distinctive electronic characteristics, reactivity and stereochemical complexity allow these compounds to engage with biomolecules and biological systems through mechanisms that are often beyond the reach of conventional organic molecules. The wide structural diversity, inherent three-dimensionality, and the ability to undergo ligand exchange, redox processes, catalysis, and photophysical interactions all contribute to the unique appeal of.

coordination complexes in medical and biotechnological contexts^1–3^. Several metal-based compounds have already achieved clinical approval across various therapeutic areas, including anti-diabetic, anti-inflammatory, anti-infective and anticancer applications^1–7^. Continued exploration of the therapeutic potential of metal chelates is likely to lead to the development of novel compounds with enhanced potency and selectivity. Moreover, amide bond formation represents one of the most fundamental transformations in organic chemistry and biochemistry, due to the widespread occurrence of amides in pharmaceuticals, natural products, and biologically active compounds^8–10^. The amide functional group is of fundamental importance in the structure of biomolecules and pharmaceuticals owing to its unique capacity to participate in hydrogen-bonding interactions^11–13^. Specifically, carbonyl oxygen can function as a hydrogen-bond acceptor, while the amine hydrogen can serve as a hydrogen-bond donor^11^. Many commercially available drugs contain amide linkages within their core structures and exhibit a wide range of therapeutic activities^8^.

Isatin (1 H-indole-2,3-dione) has attracted significant interest in medicinal and bioorganic chemistry due to its unique heterocyclic structure and its role as a valuable synthetic scaffold^14^. Isatin and its derivatives exhibited a wide range of biological activities, including antimicrobial, anticonvulsant, cysticidal, antimalarial, herbicidal, antimycobacterial, anticancer, anti-inflammatory, and antiviral effects^15–20^. Additionally, they exhibit anti-HIV, antihelminthic, and antiprotozoal activities^21^. The diverse biological activities exhibited by isatin derivatives have further reinforced its importance in drug discovery and development^14,22^. The synthetic flexibility of isatin allows for extensive structural modification through substitution, enabling the design of a wide range of derivatives targeting various biochemical pathways and molecular targets^15^.

In the current investigation, a new isatin-based Schiff base ligand (H₂L) was synthesized *via* the condensation of 4,4‘-diaminodiphenyl ether with indoline-2,3-dione. Although isatin-derived metal complexes have been widely reported, studies involving bis-isatin ligands with flexible ether linkages remain limited. To address this gap, the ligand was coordinated with VO²⁺, Ni²⁺, and Cu²⁺ ions to form the corresponding metal chelates, providing a distinct coordination environment compared to previously reported isatin–metal systems and extending their potential biological applications. The structure of the ligand and its metal chelates was comprehensively characterized using a combination of analytical and spectroscopic techniques, and quantum chemical calculations were performed to support and rationalize the experimental findings. In addition, the antidiabetic, anticancer, and antibacterial activities of the synthesized compounds were evaluated.

## Materials and methods

### Chemicals and reagents

The chemical reagents utilized in this work were sourced from Merck, Sigma Aldrich and Fluka. All the chemicals were of analytical grade and employed without any additional purification. Solvents were utilized in their original state as received.

### General procedure for the ligand preparation

**Fig. 1:**
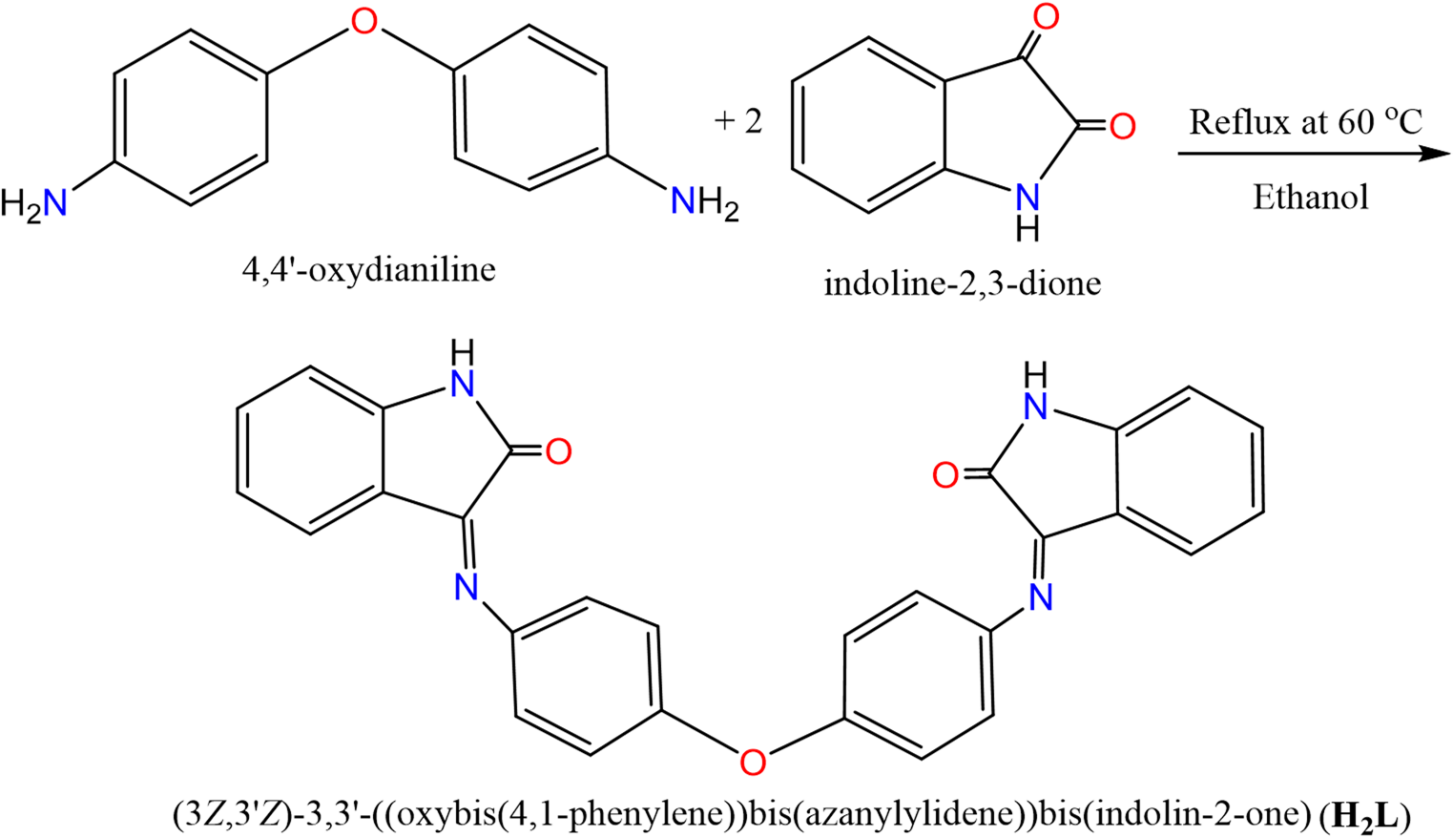
Preparation of ligand.

The newly ligand was prepared by condensing 4,4‘-diaminodiphenyl ether (0.5 g, 1.26 mmol) with indoline-2,3-dione (0.372 g, 2.53 mmol) dissolved in 20 mL absolute ethanol, in 1:2 molar ratio (Fig. 1). The reaction mixture was refluxed in round bottom flask with constant stirring at room temperature for 4 h. The thin-layer chromatography (TLC) method was used to track the reaction’s progress, visualized by UV light using hexane-acetone solution. The yellow solid formed was filtered, washed with EtOH, purified by recrystallization and dried in vacuum (actual yield 90%).

### General protocol for preparation of complexes

The metal chelates were synthesized by condensing 1 L:1 M molar ratio mixture of hot ethanolic VOSO_4_⋅H_2_O (0.1294 g, 0.71 mmol), NiCl_2_.6H_2_O (0.76 g, 3.19 mmol) or CuCl_2_.2H_2_O (0.54 g, 3.16 mmol) solution with ethanolic solution of ligand (0.4 g, 0.89 mmol), for complexes (**1–3**), respectively. Then, the refluxing process continued for 4 h. The color precipitate was filtered, rinsed, and finally evaporated under vacuum. The elemental analysis was performed on the pure product; the obtained data are given in Table 1.

**Table 1:** Analytical and physical data for the ligand and its metal chelates.

No.	Compound empirical formula	Color	Yield (%)	D.T/°C	Microanalysis Calc.(Found) %					(Λ_m_)^a^
					C	H		N	M	
**–**	(H_2_L) C_28_H_18_N_4_O_3_	Yellow	90	220	73.36 (73.52)	3.93 (4.03)	12.23 (12.31)		–	–
**1**	[(H_2_L)VO(OH)_2_].3H_2_O C_28_H_27_N_4_O_8_VO	Dark-green	90	246	54.81 (54.61)	4.24 (4.33)	9.14 (10.01)		10.92 (10.79)	10
**2**	[(H_2_L)Ni(OH)_2_] C_28_H_20_N_4_O_5_Ni	Brown	80	150	61.01 (61.30)	3.63 (3.80)	10.16 (10.20)		10.53 (10.42)	10
**3**	[LCu(H_2_O)_2_].EtOH C_30_H_26_N_4_O_6_Cu	Black	70	150	59.65 (59.70)	3.31 (3.00)	9.28 (9.40)		10.52 (10.73)	30

^a^Ω^−1^ cm^2^ mol^−1 ^DMF solution/10^− 3^mol L^− 1^.

### Analytical procedures

The procedures outlined in supplementary materials were carried out for the determination of carbon, hydrogen, nitrogen, metal and halide, magnetic susceptibilities, molar conductivity, ^1^H NMR, FT–IR, UV–Visible spectroscopy and thermal tools^23–26^.

### Computational methods

The quantum mechanical analysis was performed using density functional theory (DFT)^27,28^ with the Gaussian 09 software package^29^. Geometry optimizations, Frequency calculations, and molecular orbital generations were carried out at B3LYP functional^30–32^ with 6–31 G(d) basis set. All calculations were carried out in the gas phase. Frequency analyses were conducted on all optimized geometries at the same level of theory to confirm whether they correspond to minima or transition states on the potential energy surface of the systems. The lack of negative eigenvalues in the force-constant matrices confirms that all identified stationary points correspond to minima. The distribution of the HOMO and LUMO frontier molecular orbitals (FMOs) for both the ligand and the chelates was examined. Computational results and FMO visualizations were generated using the ChemCraft program^33^.

### Pharmacological study

#### Antidiabetic activity

The *in vitro* antidiabetic activity of the ligand and its metal chelates (**1–3**) was evaluated by α-amylase inhibition assay. The α-amylase inhibitory activity was expressed as percent inhibition and was calculated using the equation given below: The % α-amylase inhibition was plotted against the concentration of the sample and the IC_50_ values were computed from the plot. The supplemental materials provide illustrations of the procedure^34,35^.

$$\%\alpha {\text{-amylase inhibition}} = \{({\text{Abs of control}}-{\text{Abs of sample}}) \times 100\}/{\text{Abs of control}}$$

#### Cytotoxicity assays

The synthesized ligand (H_2_L) and its complexes (**1–3**) were tested for cytotoxicity against the hepatocellular carcinoma (HepG-2) cell line and also against human lung fibroblast normal cell line (WI-38) to examine the potential safety towards the normal cell line using viability assay. The cell lines (HepG-2) were obtained from the American Type Culture Collection (ATCC, Rockville, MD) and screened at the regional center for mycology and biotechnology, Al-Azhar university, Cairo, Egypt. The supplemental material provides illustrations of the anticancer screening method in detail^36,37^.

#### Antibacterial activity

Agar well diffusion method was utilized to evaluate the* in-vitro* antibacterial property of the ligand (H_2_L) and its complexes (**1–3**)^38^. The microorganisms used were *Bacillus subtilis (DSM:1088)*, *Staphylococcus aureus (ATCC:13565)* as Gram-positive bacteria and *Escherichia coli (ATCC:10536)*,* Klebsiella pneumonia (ATCC:10031)* as Gram-negative bacteria with standard drugs Ampicillin and Gentamicin. The antibacterial methodology was detailed in the supplementary materials.

## Results and discussion

### ¹H NMR spectrum of the ligand

In DMSO-*d*_*6*_ solvent, the ^1^H-NMR spectrum of the ligand (H_2_L) was recorded (Fig. 1S). ^1^H-NMR spectrum showed two peaks at 10.9 and 10.8 ppm, attributed to tautomeric equilibrium between the N–H proton (keto) and O–H proton (enol) forms in DMSO solution, with the keto form generally predominating^19^. Multiplet peaks at δ 6.63–7.36 ppm appeared in the ligand spectrum are characterized to aromatic nature of the protons (Ar-H). The peaks observed at δ 2.50 and 3.31 ppm corresponding to protons of DMSO and to the moisture present, respectively.

### Investigation of metal complexes

Table 1 showed the physical and analytical characteristics of the metal chelates. Based on the physicochemical characterization, the complexes remained non-hygroscopic, unchanged in air at room temperature and soluble in dimethylformamide (DMF) or dimethylsulphoxide (DMSO) and insoluble in most common solvents, whereas the ligand is soluble in ethanol. The data confirmed that the chelates (**1–3**) possessed 1:1 (metal-to‐ligand) stoichiometric ratio. The low conductivity values (10–30 Ω^− 1^ cm^2^ mol^− 1^) of the complexes in 10^− 3^ M DMF solvent refer to their non-electrolytic character^39^.

### Infrared spectra

The FT-IR spectra of the ligand and its metal chelates are tabulated in Table 2. The FT-IR spectrum of the free ligand showed a broad band at 3195 cm⁻¹ assignable to the N–H stretching vibration of isatin moiety. Absorption band at 1747 cm⁻¹ is characteristic of the ν(C = O)_isatin_ stretching mode. Upon complexation this band suffered lower shift of 5–42 cm⁻¹, indicating that this carbonyl group is involved in the coordination in complexes (**1**,**2**). In case of complex (**3**), the disappearance of the N–H as well as C=O stretching vibrations indicates deprotonation following enolization of (C=O)_isatin_ upon complexation, which established by the appearance of a new peak at 1247 cm⁻¹, assigned to the ν(C–O) vibration of the carbonyl group after enolization^40^. The peaks at 1612, 1464 and 1334 cm⁻¹ in the spectrum of the ligand are assigned to ν(C=N), δ(N–H) and ν(C–N)_isatin_, respectively. In all complexes, the ν(C=N) band does not alter in its shape and position upon chelation, confirming that imine groups are out of coordination. The FT-IR spectrum of the free ligand also exhibited peaks at 3462 and 1232 cm⁻¹, assignable to ν(O–H) and ν(C–O), due to the possible keto-enol forms of the ligand in the solid state^41,42^.

**Table 2:** FT**-**IR spectral bands (cm^− 1^)^a^, UV/Vis (λ_max_, nm) and effective magnetic moment data (µ_eff_, B.M.) of the compounds.

No	Compound	ν(OH/NH)	ν(C = O)	ν(C = N)	ν(M-O)	λ_max_(nm)	µ_eff_ (B.M.)
	H_2_L(C_19_H_16_N_2_O_3_)	3462(b),3195(b)	1747(s)	1612(s)	–	440/300 /275	–
**1**	[H_2_LVO(OH)_2_].3H_2_O	3447(b),3252(b),2925(b)	1742(s)	1613(s)	615(w)	810/435/300	1.82
**2**	[H_2_LNi(OH)_2_]	3428(b),3176(b),2911(b)	1705(s)	1613(b)	618(m)	990/730/440/300	3.90
**3**	[LCu(H_2_O)_2_].EtOH	3433(b),2927(w)	–	1613(b)	617(w)	550/400/285	2.27

a: s, strong; m, medium; w, weak; b, broad; ν, stretching.

In the FT-IR spectrum of the vanadyl complex (**1**), the vibration of ν(V= O) appears at 978 cm^-1^ ^[19^. Thus, the H_2_L ligand behaves as a neutral bidentate donor in complexes (1,**2**) coordinating with M^2+^ through its two carbonyl oxygen atoms, and as a bi-anionic bidentate donor, coordinating to Cu^2+^ through the deprotonated (C=O)_isatin_ after enolization in complex (**3**).

The coordination of carbonyl O is further confirmed by the appearance of new peaks in the 618–615 cm⁻¹ range, corresponding to ν(M–O) vibrations in all complexes^43,44^. Moreover, the broad absorption band at the range 3447 –2911 cm⁻¹ observed in all complexes confirms the presence of associated OH groups, whether from coordinated or lattice solvent molecules, as further supported by thermal analyses^45^.

### Magnetic measurements and electronic spectra

The stereochemistry of the metal ions in the complexes has been confirmed by using magnetic moment and electronic absorption data. The values of the maximum absorption wavelength (λ_max_, nm) and effective magnetic moment (µ_eff_ B.M.,) are shown in Table 2. The spectra of the current ligand and its metal chelates (**1–3**) were recorded within the 200–1000 nm wavelength range at room temperature in DMF.

The absorption spectrum of the ligand (H_2_L) showed characteristic bands at 440, 300 and 275 nm ascribed to charge transfer (CT), n–π* and π-π* transitions, respectively.

The electronic spectrum of VO^2+^ complex (**1**) displayed a new absorption band at 810 nm which ascribed to ^2^B_2_(*d*_*xy*_)→^2^E(*d*_*xz*_,*d*_*yz*_) transitions, in addition, the FT–IR spectrum exhibited the ν(V=O) at 978 cm^− 1^ indicating square-pyramidal geometry^46^. The measured magnetic moment value of VO^2+^ complex (**1**) is 1.82 B.M. which indicated the existence of VO^2+^ in square-pyramidal geometry^47^.

Two new absorption peaks at 990 and 730 nm assigned to d–d transitions; ^3^A_2g_ (F)→^3^T_2g_ (F) ʋ_1_, ^3^A_2g_ (F)→^3^T_1g_(F) ʋ_2_ has been recorded for Ni^2+^ complex (**2**), confirming the tetrahedral geometry around Ni^2+^ ion. The µ_eff_ value of this complex is 3.9 B.M. suggesting high spin tetrahedral geometry^48^.

Cu^2+^ complex (**3**) displayed a broad peak centered at 550 nm, assignable to ^2^B_1g_→^2^A_1g_, which indicated that the Cu^2+^ complex has distorted square planar geometry^49^. The magnetic moment of 2.27 B.M. is within the typical range observed for one unpaired electron^50^.

### Thermal analysis studies

**Fig. 2:**
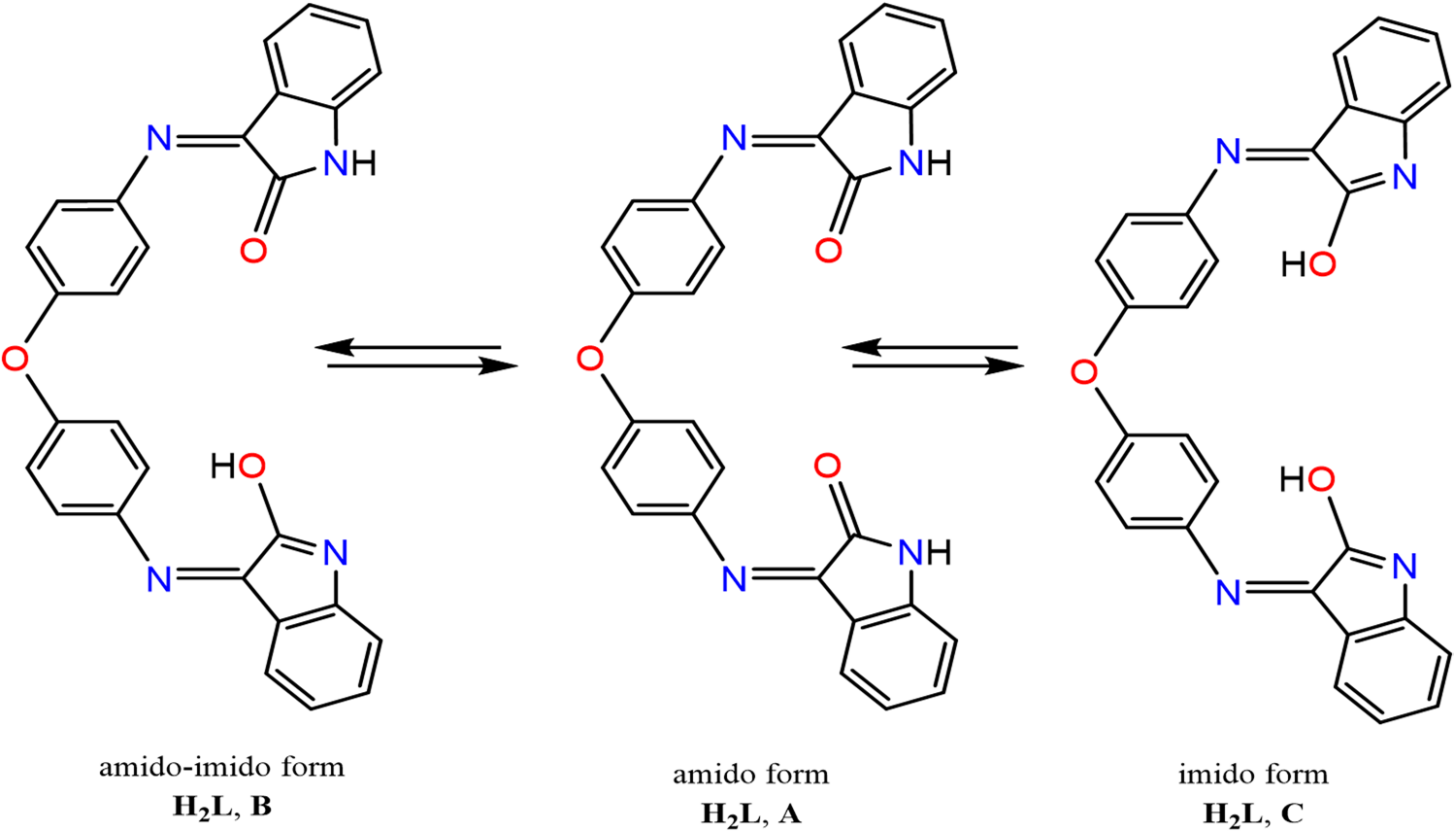
Amido–imido tautomers of H_2_L ligand.

Thermogravimetric (TGA/DTG) technique has been performed in order to identify whether the H_2_O molecules are of crystallization or coordinated to the central metal ion and to give additional details about the structure and thermal degradation of formed new compounds. The thermogravimetric studies have been conducted between room temperature and 800 °C. The data was displayed in Table 3 and shown in Fig. 2S.

**Table 3:** TGA analysis of the metal complexes.

No.	Compound	Temp. range /°C		Mass loss%		Leaving species
		DTG	TG	Calc.	(Found)	
**1**	[H_2_LVO(OH)_2_].3H_2_O C_28_H_27_N_4_O_8_VO	61	30–150	8.80	8.71	-3H_2_O
		236	150–250	5.54	(5.60)	-2(OH)
		310,362	250–450	72.16	(72.10)	-C_28_H_19_N_4_O_3_
			at 500	13.50	(13.59)^a^	≡VO_2_
**2**	[H_2_LNi(OH)_2_] C_28_H_20_N_4_O_5_Ni	54	30–150	–	–	Steadly stable
		274	150–280	6.17	6.20	-2OH (decomp)
		366	280–450	47.12	(47.30)	-C_16_H_8_N_2_O_2_
			450–655	36.07	(35.90)	-C_12_H_12_N_2_O
			at 655	10.64	(10.60)^a^	≡Ni
**3**	[LCu(H_2_O)_2_].EtOH C_30_H_26_N_4_O_6_Cu	50	30–100	7.62	(7.60)	-EtOH
		202	150–225	6.15	(6.20)	-2H_2_O
		372	225–644	75.71	(75.87)	-C_28_H_16_N_4_O_3_
			at 644	10.52	(10.33)^a^	≡Cu

^a^Final product percent.

The TG and DTG curves of [H_2_LVO(OH)_2_].3H_2_O complex (**1**) showed weight loss (Calc./Found %); (8.80/8.71) representing loss of hydrated 3H_2_O molecules within the 30–150°C range, associated with maximum one DTG band at 61°C, then TG curve showed loss of two coordinated OH group at 150–250°C (Table 3). The TG curve also, showed loss of the ligand’ organic part within 250–450 °C, with mass loss (Calc./Found %); (77.16/77.10), accompanying with DTG peaks at 310,362 °C. Vanadium oxide as the ultimate pyrolysis products was obtained at 500 °C (Table 3).

The TG and DTG curves of [H_2_LNi(OH)_2_] complex (**2**) exhibited steady part till 150 °C. On further heating, the decomposition of the complex starts at 150 °C (Calc./Found %); (6.17/6.20) congruent with the loss of 2OH groups. At 280–450 °C range the curve showed weight loss (Calc. 47.12/Found 47.3%), corresponding to elimination of C_16_H_8_N_2_O_2_ part. The removal of organic part is completed at 655 °C, with weight loss (Calc./Found %); (36.07/35.90) corresponding to C_12_H_12_N_2_O moiety, with DTG peaks at 366 °C. Finally, Ni metal as the ultimate pyrolysis product was obtained (Table 3).

The TG and DTG curves of [LCu(H_2_O)_2_].EtOH complex (**3**) showed weight loss (Calc./Found %); (7.62/7.60) from room temperature up to 100 °C, associated with one endothermic DTG band at 50 °C, congruent with the loss one ethanol molecule. From 150 to 225 °C the TG curve showed weight loss (Calc./Found %); (6.15/6.20) corresponding to removal of two coordinated 2H_2_O molecules. Finaly, the organic part decomposed in the 225–644 °C range with DTG peaks at 202,372 °C, with final decomposition residue as Cu metal (Table 3).

### DFT studies

**Fig. 3:**
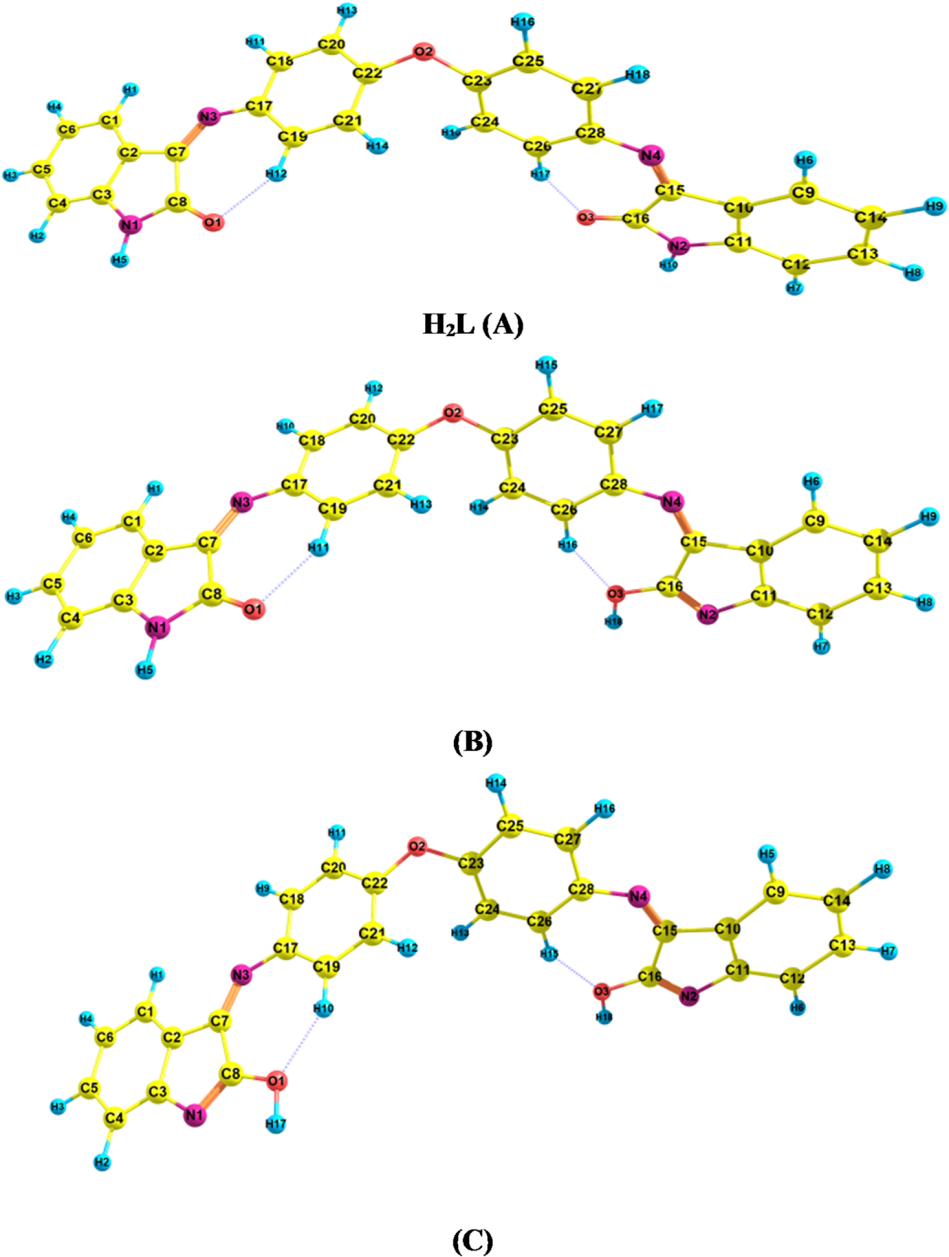
Optimized structures of the ligand (H_2_L**)** tautomers.

In view of the unsuccessful attempts to obtain crystalline samples suitable for X-ray crystallographic analysis, density functional theory (DFT) calculations were undertaken to achieve a more comprehensive understanding of the metal–ligand bonding in the selected complexes. The geometries of the ligand tautomers and their corresponding complexes (**2** and **3**) were fully optimized at the DFT(B3LYP)/6-31G(d) level of theory, considering a doublet spin state (multiplicity = 2) for the Cu^2+^ complex (**3**) and a triplet spin state (multiplicity = 3) for the Ni^2+^ complex (**2**) (Figs. 2 and 3). Subsequently, FT-IR vibrational frequency calculations were performed, and the absence of any imaginary frequencies confirmed that all optimized structures correspond to true minima on their respective potential energy surfaces. Furthermore, the thermodynamic parameters, including the total electronic energy (E), enthalpy (H = E + PV), entropy (S), and Gibbs free energy (G = H – TS, at T = 298.15 K), were computed at the same level of theory to provide additional insight into the thermodynamic stability of the studied systems.

#### Geometrical optimization of the ligand

The ligand (H_2_L) is capable of existing in multiple tautomeric forms, specifically the amido (keto) and imido (enol) configurations, as illustrated in Fig. 2. The optimized structures of these tautomers are presented in Fig. 3. Among the calculated forms, the diketo tautomer (form A) exhibits the lowest total energy, indicating that it represents the most stable form in the gas phase (Table 4). Furthermore, the amido–imido tautomer (form B) was found to be thermodynamically more stable than the dienol tautomer (form C). Theoretical results therefore suggest that the ligand predominantly exists in the diketo configuration (form A), which is in good agreement with the experimental observations. The DFT calculations also revealed that, in the gas phase, the keto form (A) is more stable than the enol form (C) by approximately 52 kcal·mol⁻¹, further confirming the preference for the diketo tautomeric form. To further elucidate the electronic structures of the ligand and its metal complexes, a natural bond orbital (NBO) analysis was carried out. The atomic charges were obtained through natural population analysis (NPA), and the calculated NPA charges for selected atoms of the ligand and its complexes are summarized in Table 5. The results indicate that the oxygen atoms (O1 and O2) possess higher negative charges (–0.513) compared to the nitrogen atoms (N3 and N4) (–0.377). Consequently, it can be inferred that the metal ion exhibits a stronger binding affinity toward the oxygen atoms rather than the nitrogen donors. The selected geometrical parameters for the free ligand (H₂L) are listed in Table 6.

**Table 4:** The relative energies (kcal/mol) calculated for different tautomers of the ligand (H_2_L) at B3LYP∕ 6-31G(d).

Tautomer	∆E	∆E_0_	∆E_298_	∆H_298_	∆G_298_
**A**	0	0	0	0	0
**B**	26.30	25.27	25.40	25.40	24.96
**C**	52.73	50.67	50.93	50.93	50.09

**Table 5:** NBA charges of the ligand and the studied complexes at B3LYP∕ 6-31G(d).

Atom/compd	H_2_L (A)	B	C	2	3
**O1**	− 0.513	− 0.514	− 0.619	− 0.560	− 0.590
**O3**	− 0.513	− 0.619	− 0.619	− 0.549	− 0.590
**N1**	− 0.627	− 0.625	− 0.604	− 0.617	− 0.617
**N2**	− 0.625	− 0.494	− 0.604	− 0.622	− 0.617
**N3**	− 0.377	− 0.378	− 0.374	− 0.340	− 0.356
**N4**	− 0.377	− 0.374	− 0.374	− 0.334	− 0.356
**M**	–	–	–	0.935	0.976

**Table 6:** Calculated binding energies (kcal/mol) for the studied complexes in the gas phase at B3LYP∕ 6-31G(d).

Compd	∆E_0_	∆E_298_	∆H_298_	∆G_298_
**2**	− 1545.87	− 1546.79	− 1549.16	− 1516.55
**3**	− 42.56	− 44.47	− 46.24	− 10.929

The energy gap between the HOMO and LUMO levels provides key insights into a molecule’s chemical stability, reactivity, and hardness^42,45^. Hardness is a particularly valuable concept for interpreting the behavior and reactivity of chemical systems. It is closely related to properties such as softness, electronic chemical potential, and absolute electronegativity^51,52^. While hardness reflects molecular stability, softness is indicative of chemical reactivity. Based on the calculated HOMO and LUMO energy values, several global reactivity descriptors—including the energy gap (ΔE), absolute electronegativity (χ), chemical potential (µ), absolute hardness (η), absolute softness (σ), global electrophilicity index (ω), global softness (S), and maximum electronic charge transfer (ΔN_max)—were evaluated to assess the stability and reactivity of the ligand and its corresponding metal complexes. The computed parameters are summarized in Table 7. The results reveal that the free ligand possesses a relatively small HOMO–LUMO energy gap (3.23 eV), indicating higher softness and enhanced chemical reactivity toward metal ion coordination. Furthermore, the high electrophilicity index (ω) value suggests that the ligand may exhibit significant biological activity.

#### Structure of the complexes

**Fig. 4:**
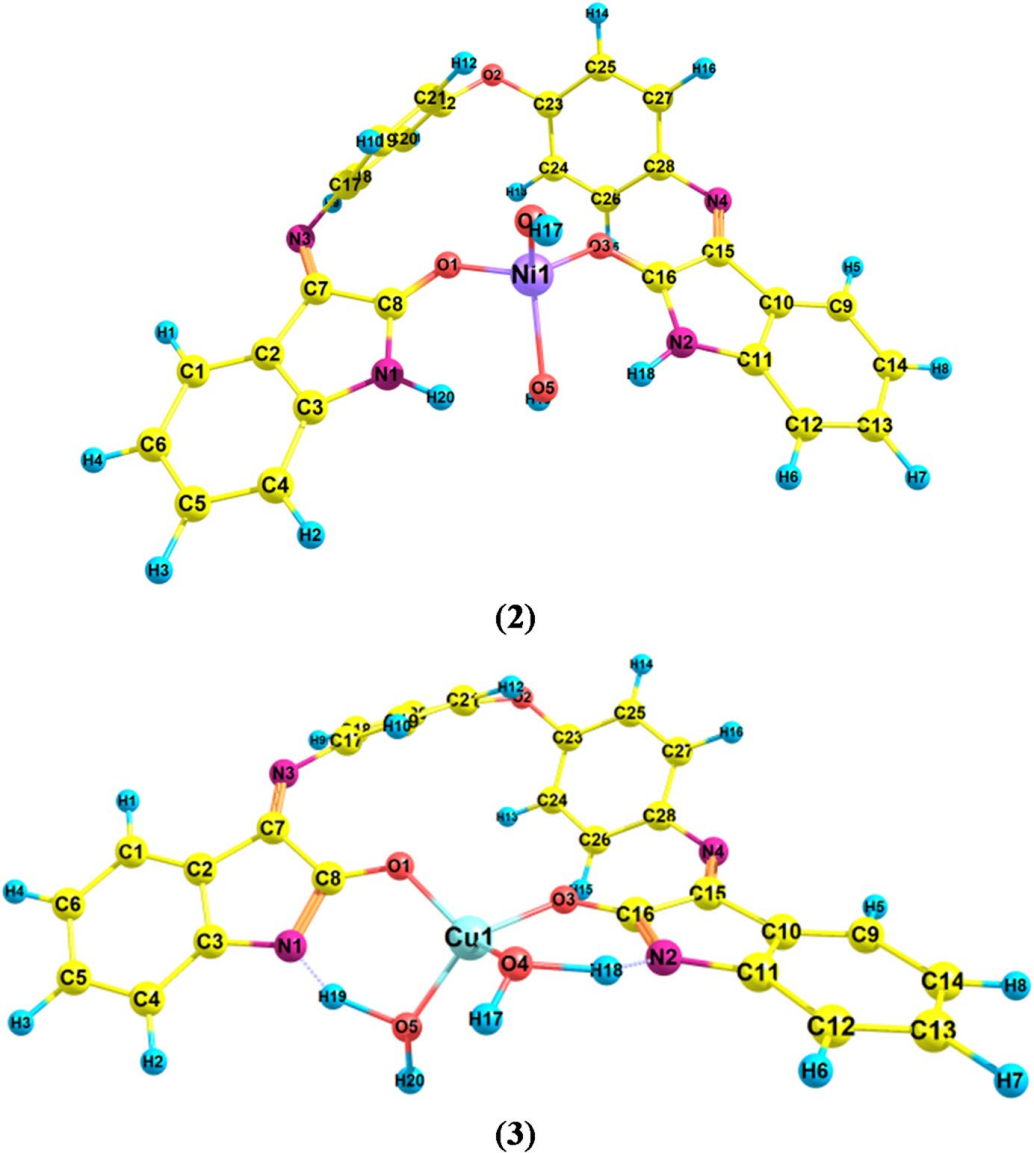
Optimized structures of the studied complexes (**2**, **3**).

The optimized geometries of the studied complexes (**2** and **3**) are illustrated in Fig. 4. Complex (**2**) exhibits a tetrahedral geometry, whereas complex (**3**) adopts a distorted square-planar configuration. In both complexes, the ligand coordinates to the metal center in a bidentate fashion via the two oxygen donor atoms (O1 and O2). In the Ni²⁺ complex (**2**), the metal ion preferentially coordinates with the oxygen atoms (O1 and O2) in the keto form of the ligand. In contrast, in the Cu²⁺ complex (**3**), deprotonation following enolization is essential to facilitate coordination of the metal ion with the ligand.

On the other side, the calculated binding energies of the complexes in the gas phase are summarized in Table 8. The negative values of these binding energies confirm that the corresponding complexation reactions are thermodynamically favorable. The selected bond lengths for the optimized complexes are presented in Table 6. The results reveal that the Cu–O (enolic) bond lengths are shorter than the corresponding Ni–O (ketonic) bond distances, which can be attributed to the greater ionic character and stronger metal–oxygen interaction in the copper complex. Furthermore, the negative charges on the oxygen atoms O1 and O2 increase upon complexation in complexes (**2**) and (**3**) relative to the free ligand, indicating the occurrence of back-donation from the metal center to the ligand oxygen atoms. In addition, the metal ions in both complexes possess charges less than + 1 e, reflecting significant charge transfer from the ligand to the central metal ion. The calculated dipole moments (D) of the complexes range from 3.68 to 5.38 Debye (Table 7), confirming that these species exhibit a polar nature.

**Table 7:** Selected bond lengths (B, Ǻ) for the complexes at B3LYP∕ 6-31G(d).

	C8-O1	C8-N1	C16-O3	C16-N2	M-O1	M-O3	M-O4	M-O5
H_2_L (A)	1.239	1.393	1.239	1.393	–	–	–	–
**B**	1.239	1.392	1.362	1.296	–	–	–	–
**C**	1.362	1.296	1.362	1.296	–	–	–	–
**2**	1.264	1.360	1.262	1.361	1.918	1.919	1.743	1.944
**3**	1.280	1.349	1.280	1.349	1.909	1.909	1.835	1.835

**Table 8:** Various quantum chemical parameters of the free ligand and the complexes at B3LYP∕ 6-31G(d).

	(A) H_2_L	B	C	2	3
Dipole moment (D)	2.3215	3.6540	0.4956	5.3815	3.6879
E_HOMO_ (eV)	− 5.69	− 5.82	− 5.98	− 4.71	− 5.50
E_LUMO_ (eV)	− 2.45	− 2.55	− 2.65	− 2.79	− 2.53
ΔE (eV)	3.23	3.27	3.33	1.92	2.97
χ (eV)	4.070	4.185	4.335	3.75	4.015
η (eV)	1.620	1.635	1.665	0.96	1.485
σ (eV^− 1^)	0.617	0.611	0.600	1.041	0.673
Pi (eV)	− 4.070	− 4.185	− 4.335	− 3.75	− 4.015
S (eV^− 1^)	0.308	0.305	0.300	0.520	0.336
ω (eV)	5.112	5.356	5.643	7.324	5.427
ΔN_max_	2.512	2.559	2.603	3.906	2.703

**Fig. 5:**
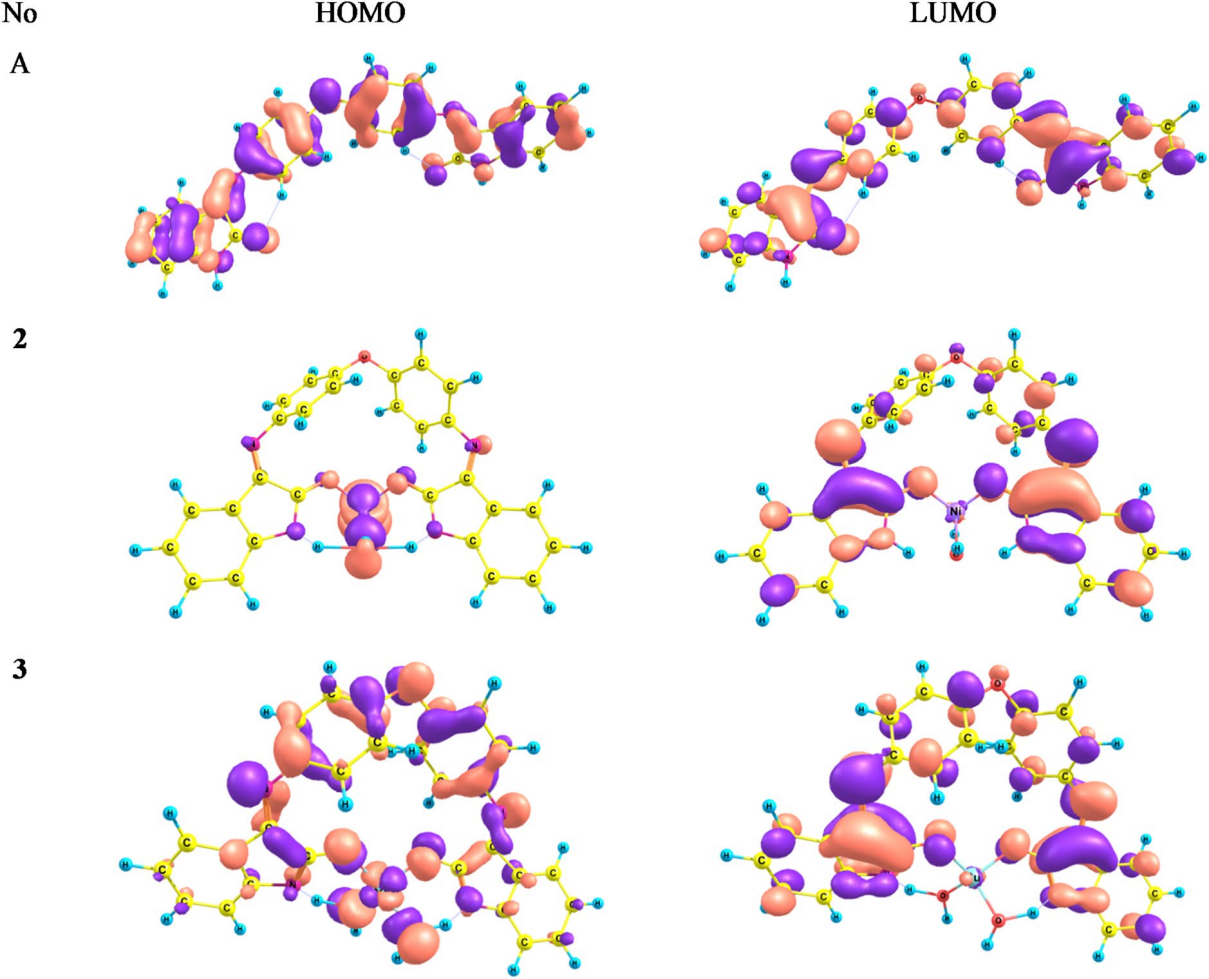
DFT computed HOMO and LUMO diagrams of the ligand and its complexes.

Based on the calculated HOMO and LUMO energies, various chemical reactivity parameters for the studied compounds were evaluated and are summarized in Table 7. The HOMO energies of complexes (**2**) and (**3**) are destabilized by 0.19 and 0.98 eV, respectively, while the LUMO energies are stabilized by 0.34 and 0.08 eV relative to the free ligand, resulting in a decrease in the HOMO–LUMO energy gap in the complexes (Table 7). Furthermore, the HOMO electron density in the complexes is delocalized over both the metal center and its coordination sphere, reflecting the strong metal–ligand interactions (Fig. 5). The calculated frontier molecular orbital parameters reveal a noticeable decrease in the HOMO–LUMO energy gap upon coordination, particularly for the Ni^2+^ complex (**2**), which exhibits the smallest ΔE value (1.92 eV) compared with the free ligand system (ΔE ≈ 3.23–3.33 eV). This significant reduction in the energy gap indicates facilitated electronic excitation and enhanced charge-transfer character, which is consistent with the appearance of new low-energy absorption bands at 990 and 730 nm in the electronic spectrum of complex (**2**). Similarly, the Cu^2+^ complex (**3**) shows a reduced ΔE value (2.97 eV) relative to the ligand, correlating well with the observed bathochromic shift and the broad absorption band centered at 550 nm. In contrast, the free ligand (H₂L), with a larger HOMO–LUMO gap, displays higher-energy transitions in the UV region (275–440 nm). Overall, the decrease in the HOMO–LUMO gap upon complex formation supports the experimentally observed red shift and the emergence of d–d and charge-transfer transitions in the metal complexes.

In addition, the calculated global reactivity descriptors further support the experimental observations. Upon complexation, a decrease in global hardness (η) and a corresponding increase in global softness (S) are observed for the Ni^2+^ and Cu^2+^ complexes compared with the free ligand. Increased molecular softness is commonly associated with enhanced chemical reactivity and adaptability toward biological environments. Accordingly, the higher softness and electrophilicity values of the Ni^2+^ and Cu^2+^ complexes are consistent with their enhanced α-amylase inhibitory and antibacterial activities compared with the ligand. The electrophilicity index (ω) is a useful descriptor for assessing the biological activity and potential toxicity of compounds, as a linear correlation has been observed between electrophilicity and molecular bioactivity^53^. Notably, the complexes exhibit higher electrophilicity values compared to the free ligand, suggesting that the studied complexes may possess enhanced biological activity relative to the uncoordinated ligand.

It should be noted that no DFT calculations were performed for the VO^2+^ complex (**1**); therefore, its biological and spectroscopic behavior is discussed exclusively based on experimental measurements. Overall, the computational results complement the experimental findings by rationalizing the observed spectroscopic and biological activity trends rather than establishing direct quantitative correlations.

**Fig. 6:**
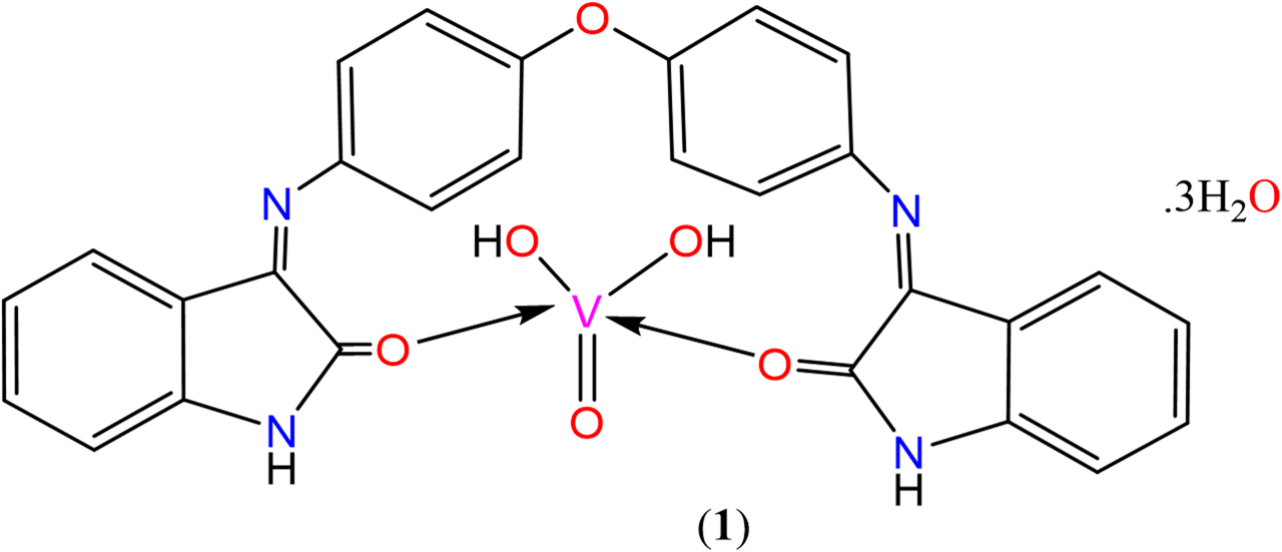
Structure of complex (**1**).

The proposed chemical structure of VO^2+^ complex (**1**) from the aforementioned assignment is presented in Fig. 6.

### Biological studies

#### Antidiabetic assay

**Fig. 7:**
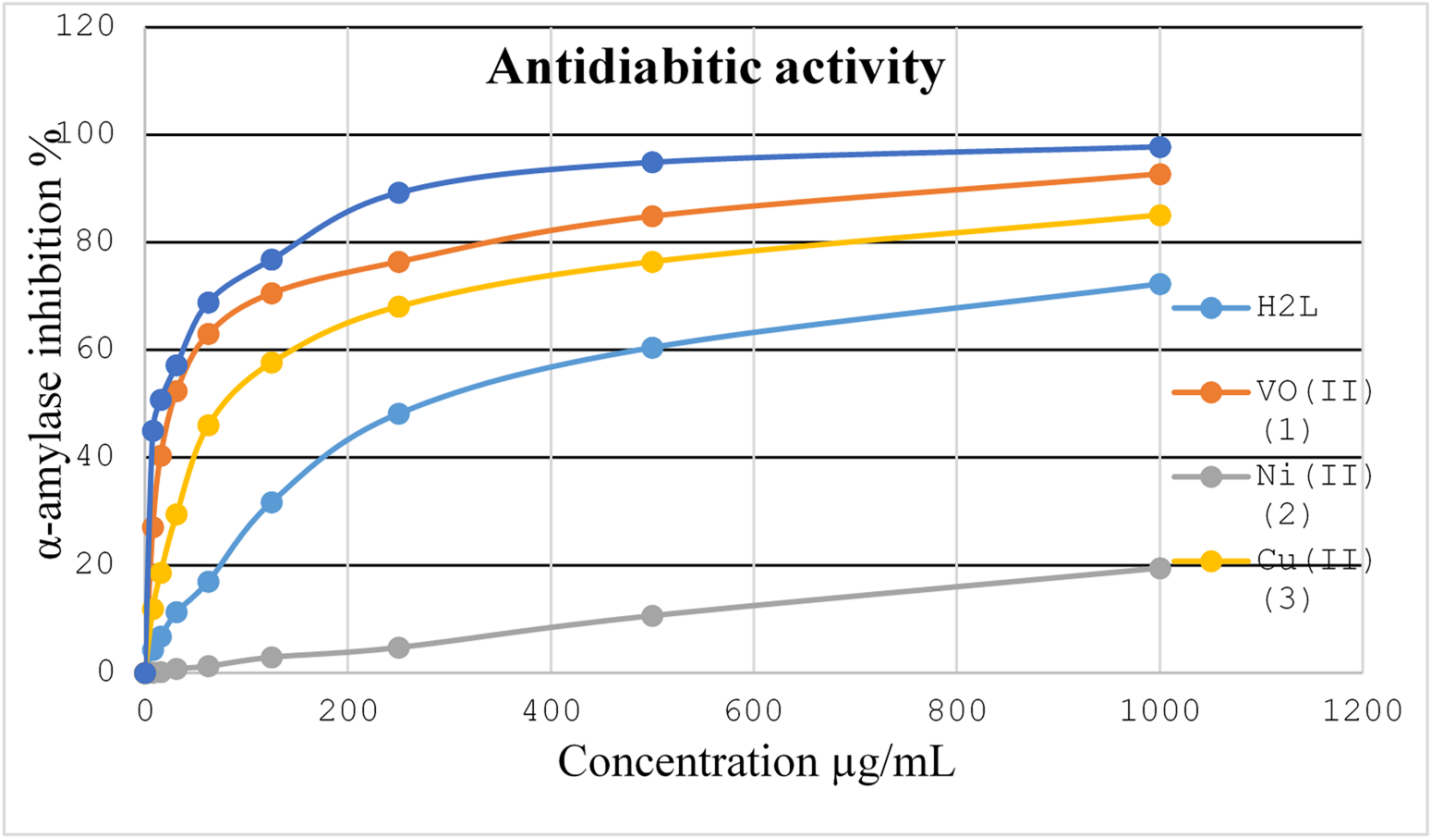
Inhibitory effects of the ligand and its metal(II) chelates (**1**,**2.3**) on α-amylase.

**Fig. 8:**
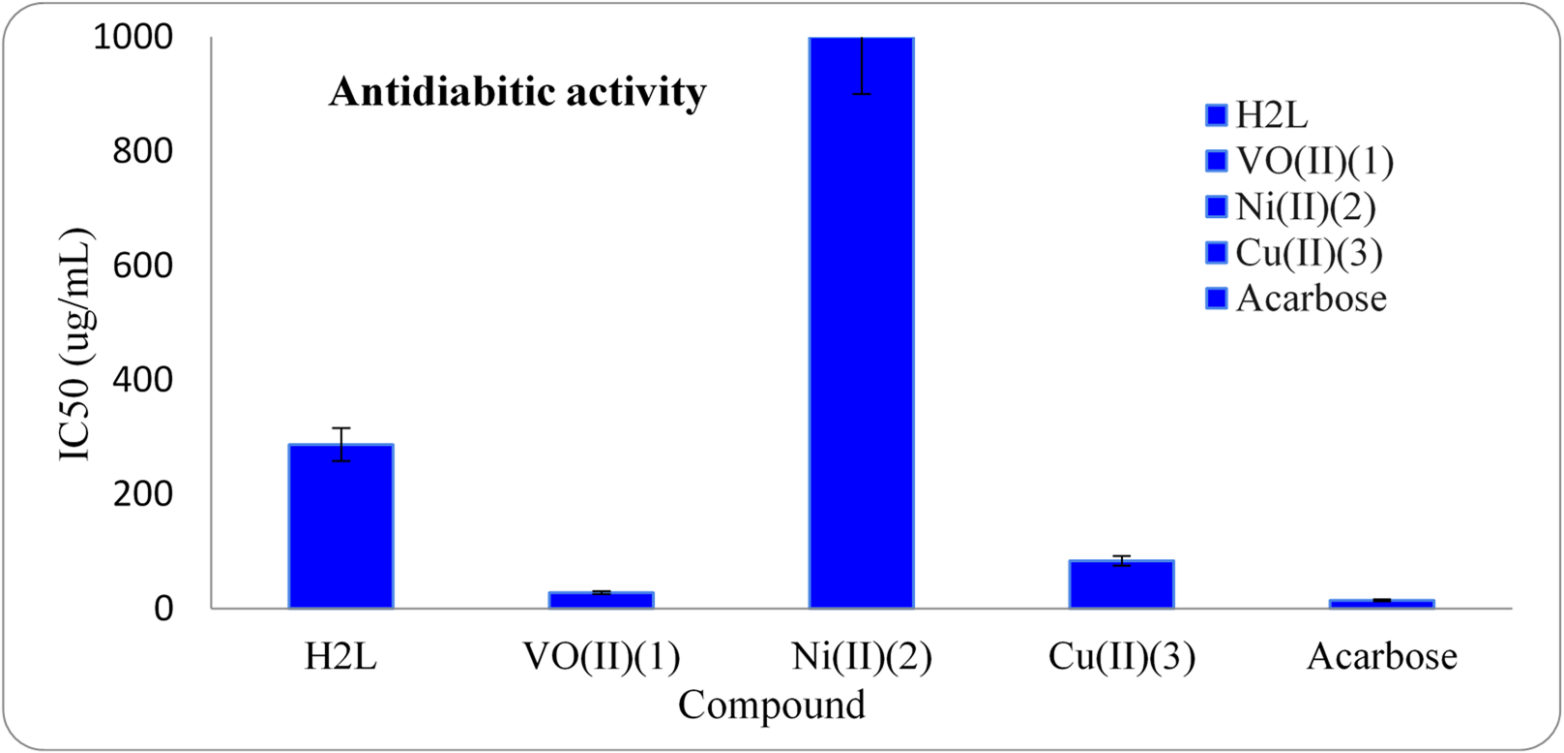
IC_50_ values for the α- amylase inhibitory activity of the ligand and its metal(II) chelates (**1**,**2.3**) on α-amylase.

Reduced insulin secretion and increased hepatic glucose production cause diabetes mellitus^54^. The digestive enzyme α-amylase is accountable for maltose hydrolysis, a dietary starch, leading to glucose production. It is essential to control the activity of α-amylase to prevent postprandial hyperglycemia (PPHG) in diabetic conditions^55^. Unregulated diabetes can result in numerous chronic disorders, such as blindness, kidney and heart failure. The *in vitro* antidiabetic activity of the current ligand and its chelates (**1–3**) was evaluated using α-amylase enzyme inhibitory method. It has been tested towards the action of α-amylase at varying concentrations. However, for comparison, standard drug acarbose inhibitory property has also been studied at the same concentrations (Fig. 7). The results of IC_50_ (which represents the concentration of an inhibitor required to inhibit 50% of the enzyme activity, lower IC_50_ indicates greater potency of the inhibitor) for the ligand and its metal chelates are tabulated in Table 9 and depicted in Fig. 8. The IC_50_ value for the ligand is found to be 286.82 µg/mL. VO^2+^ complex (**1**) displayed the IC_50_ value equals 28.13 µg/mL compared to that of the reference acarbose (14.53 µg/mL), followed by Cu^2+^ complex (**3**) that has IC_50_ value of 83.78 µg/mL. However, the Ni^2+^ complex (**2**) almost showed no α-amylase inhibitory activity (IC_50_ >1000 µg/mL). Here from the results, VO^2+^ complex (**1**) possesses highest inhibitory activities on α-amylase followed by Cu^2+^ complex (**3**). It has been noted that the α-amylase inhibitory property of the synthesized ligand improved upon chelation with VO^2+^ and Cu^2+^ ions. The enhanced activity of VO^2+^ and Cu^2+^ complexes might be assigned to the enzyme deactivation *via* complexation. Significant factors in inhibition may be the type of metal ion and its coordination geometry, that lead to improved interaction between the metal complex and the enzyme’s active sites. Additionally, by generating the transfer of glucose into peripheral tissue cells, the metal complexes used may lower blood glucose levels. Based on the findings, VO^2+^ complex (**1**) possesses excellent inhibitory properties on α-amylase and can be suggested for further antidiabetic studies to discover its medicinal application^56,57^.

**Table 9:** IC_50_ values for the α-amylase inhibitory activity of the ligand and its chelates **(1–3)**.

No	Compound	IC_50_(µg/mL) α-amylase
**–**	H_2_L	286.82 ± 6.87
**1**	[H_2_LVO(OH)_2_].3H_2_O	28.13 ± 0.65
**2**	[H_2_LNi(OH)_2_]	> 1000
**3**	[LCu(H_2_O)_2_].EtOH	83.78 ± 3.64
**–**	Acarbose	14.53 *±* 0.81

#### The cytotoxicity efficiency of H_2_L and its metal chelates

**Fig. 9:**
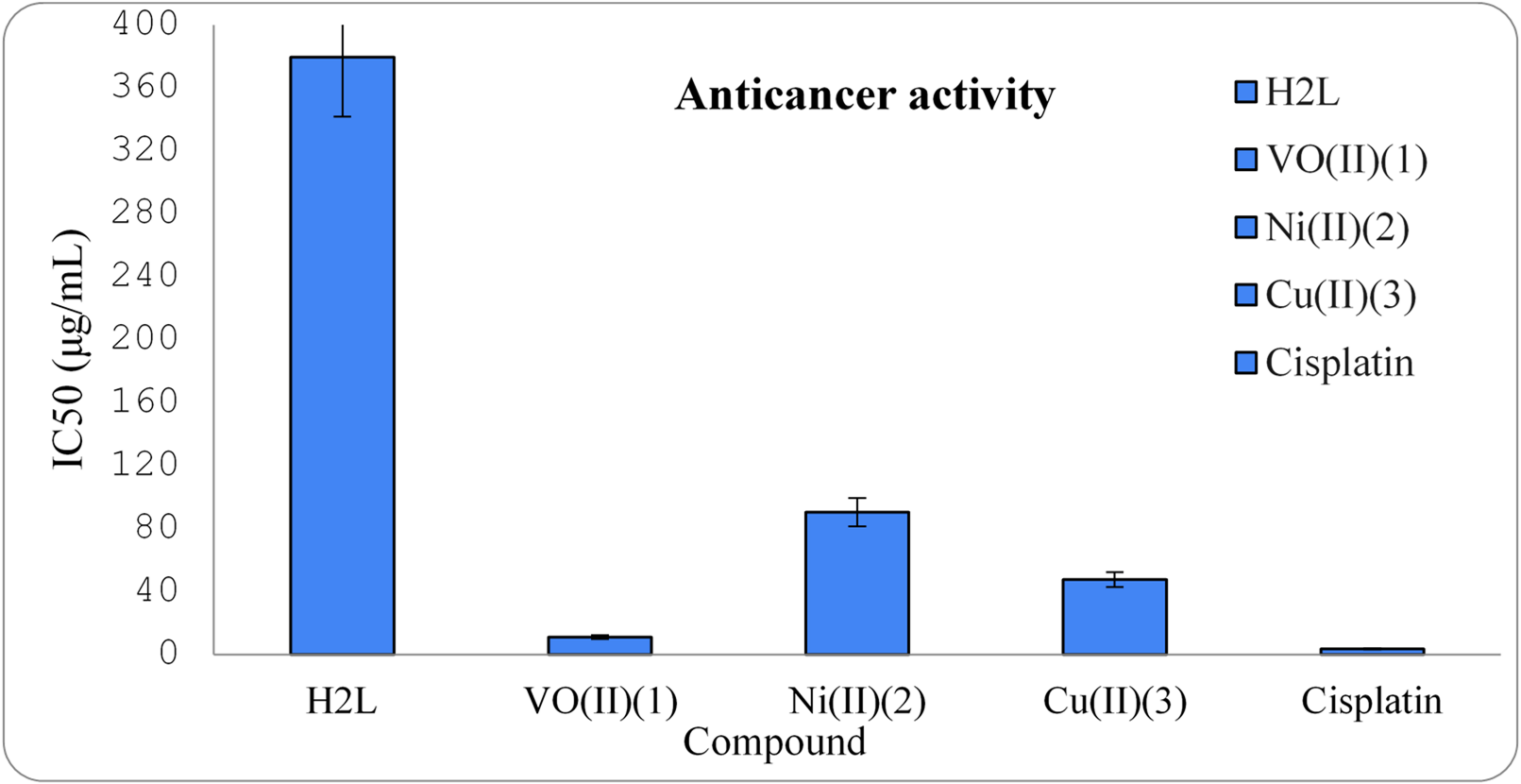
Anticancer activity for ligand and its chelates (**1**,**2**,**3**) on HepG-2.

The cytotoxic activity of the synthesized compounds; H_2_L and its chelates (**1–3**) were examined *in vitro* on HePG-2 (hepatic cancer cell line) by viability assay method, along with the standard Cisplatin for comparison purposes. The cytotoxic effect is quantified as the IC_50_ value (concentration needed to reduce 50% of the cancer cells). The findings are depicted in Fig. 9 and the IC_50_ values are mentioned in Table 10. The obtained data indicated that the tested complexes (**1–3**) showed better activity than their metal free ligand. The data also showed that [H_2_LVO(OH)_2_].3H_2_O complex (**1**) possesses strong anticancer activity (IC_50_ =11.13 ± 0.32 µg/mL) towards HePG-2 cell line followed by [LCu(H_2_O)_2_].EtOH (**3**) that possesses moderate activity (IC_50_ =47.57 ± 1.34 µg/mL). Conversely, [H_2_LNi(OH)].H_2_O complex (**2**) exhibited weak toxicity with IC_50_ value of (IC_50_ =90.56 ± 2.91 µg/mL), and the ligand possessed no activity (IC_50_ >100 µg/mL) compared to cisplatin (IC_50_ = 3.57 ± 0.21 µg/mL). The overall *in vitro* sequence against HePG-2 of the observed cytotoxicity is:

Cisplatin > VO^2+^ (**1**) > Cu^2+^ (**3**) > Ni^2+^ (**2**) > H_2_L.

**Table 10:** Lethal concentration (IC_50_) of the ligand and its chelates (1–3) on HepG-2 and cell cytotoxic concentration (CC_50_) of the ligand and its Ni(II) complex (**2**) on human lung fibroblast (WI-38).

No.	Compound	IC_50_(µg/mL)^a^ HepG-2	CC_50_(µg/mL)^b^ WI-38
**–**	H_2_L	379.71 ± 9.87	436.37 ± 10.95
**1**	[H_2_LVO(OH)_2_].3H_2_O	11.13 ± 0.32	–
**2**	[H_2_LNi(OH)_2_]	90.56 ± 2.91	181.687.82
**3**	[LCu(H_2_O)_2_].EtOH	47.57 ± 1.34	–
**–**	Cisplatin	3.57 ± 0.21	–

^a^IC_50_ value is the concentration at which 50% survival of cells was observed.

^b^The 50% cell cytotoxic concentration.

The *in vitro* property of anticancer agents can be linked to their lipophilic property; more hydrophobicity can cause increasing in the uptake of the substance by the cells, hence, enhanced the anticancer activity. By comparison of the anticancer property of similar complexes in some other work reported earlier, we found an enhancement in the property of the current compounds under investigation towards the selected cell^6,19,44,50^. Several other aspects such as molecular structure, different metal ion, steriostructure, steric properties, particle size of the metal ion, solubility can impact on the biological efficiency^58^.

The cytotoxic property of the ligand (H_2_L) and its [H_2_LNi(OH)_2_] complex (**2**) against human lung fibroblast normal cell line (WI-38) cells was also evaluated to explore the potential safety towards the normal cell line. The results were calculated and given (Table 1S) with the 50% cell cytotoxic concentration CC_50_ = 436.37 ± 10.95, 181.687.82 µg/mL for the ligand and its Ni^2+^ (**2**) complex, respectively. The viability of the compounds represented in relation to their concentration in Fig. 3S. The findings indicated that the tested compounds were less cytotoxic to normal (WI-38)) cells. This implies their anticancer potential^6^.

#### Antibacterial property

**Fig. 10:**
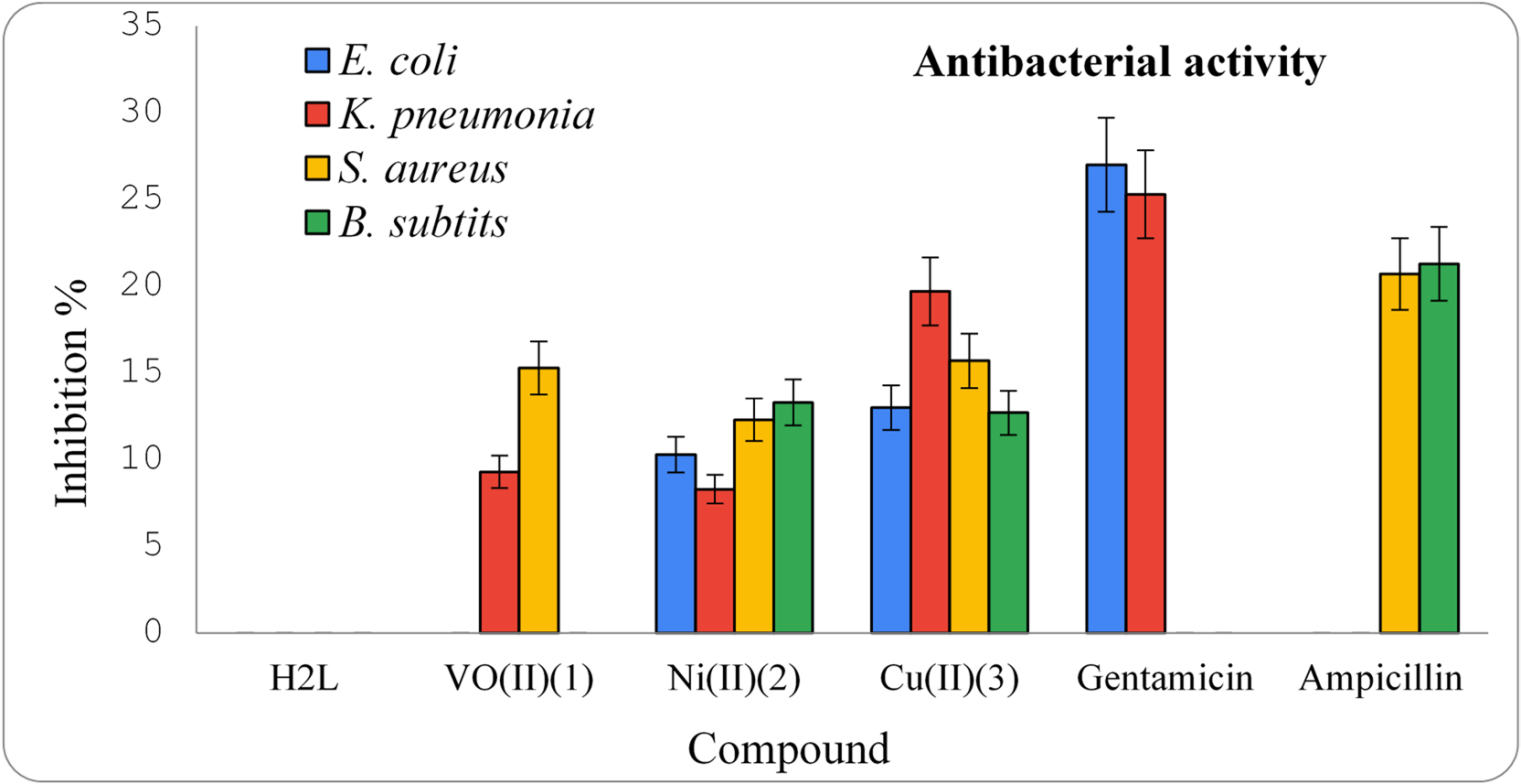
Antimicrobial activity for ligand and its chelates (**1**,**2**,**3**).

The *in-vitro* antibacterial efficacy of the investigating ligand and its metal chelates (**1–3**) was assessed towards two Gram-positive (+ ve) bacteria (*B. subtilis* and *S. aureus*) and two Gram-negative (-ve) bacteria (*E. coli* and *K. pneumonia*). The results obtained of the antibacterial activities against the tested bacteria along with standard drugs: Ampicillin (Gram +ve) and Gentamicin (Gram -ve) are represented in Table 1S and the corresponding representative graph is depicted in Fig. 10. The ligand had no activity on both Gram +ve and Gram -ve bacterial strains. In general, the chelates showed better antibacterial property than the metal free ligand. The chelates (**1–3**) showed significant activity against *S. aureus* (Gram-positive): 15.3 ± 0.6, 12.3 ± 0.6 and 15.7 ± 0.6 mm, respectively, compared with the standard drug Ampicillin (20.7 ± 0.6 mm). The complexes (**2**,**3**) showed moderate activity against *B. subtilis*: 13.3 ± 0.6 mm for complex (**2**) and 12.7 ± 0.6 mm for complex (**3**) whereas complex (**1**) has no activity compared with the reference Ampicillin (21.3 ± 0.6 mm).

With respect to (Gram-negative) *E. coli* strain, complexes (**2**,**3**) showed activity values: 10.3 ± 0.6 mm for complex (**2**) and 13.0 ± 1.0 mm for complex (**3**), while complex (**1**) has no activity, compared with the standard drug Gentamicin (27.0 ± 1.0 mm). With respect to *K. pneumonia*, the complexes (**1–3**) showed activity values: 9.3 ± 0.6 mm for complex (**1**), 8.3 ± 0.6 mm for complex (**2**) and 19.7 ± 0.6 mm for complex (**3**), respectively, compared with the standard drug Gentamicin (25.3 ± 0.6 mm). It is worth noting that, the Cu^2+^ complex (**3**), is the most potent antibacterial agent and possesses the highest inhibition zone towards *S. aureus*,* E. coli* and *K. pneumonia.* The antibacterial activities of the screened ligand and its chelates can be arranged as follows:

Ampicillin > Cu^2+^ (**3**) > VO^2+^ (**1**) > Ni^2+^ (**2**) > H_2_L For *S. aureus*.

Ampicillin > Ni^2+^ (**2**) > Cu^2+^ (**3**) > VO^2+^ (**1**) = H_2_L For *B. subtilis*.

Gentamicin > Cu^2+^ (**3**) > Ni^2+^ (**2**) > VO^2+^ (**1**) = H_2_L For *E. coli*.

Gentamicin > Cu^2+^ (**3**) > VO^2+^ (**1**) > Ni^2+^ (**2**) > H_2_L For *K. pneumonia*.

The increased activity of the metal(II) complexes implied that complexation might facilitate the compounds’ passage* via *a cell membrane and can be discussed by Tweedy’s chelation theory^59^. When metal ions bind to ligand, it decreases the polarity of the metal atom by partially sharing its positive charge with donor groups and electron delocalization over the entire chelate. Chelation could make the central metal atom more lipophilic, which would enhance its ability to pass through the cell membrane’s lipoid layer and enable the metal complex to more successfully penetrate the bacterial membrane, leading to increase the activity of the compounds^60^. This may be owing to the greater lipophilic nature of the chelates. The inactivity of the ligand and VO^2+^ Complex (**1**) may attribute to their inability to penetrate into the bacterial cell membrane and so not able to inhibit its bacterial potency. In addition, many reasons such as solubility, complex geometry, type of metal ions’ size and charge, bond length, dipole moment, concentration, steric hindrance factors, conductivity and coordination sites play an important effect on the antibacterial performance of these chelates^61–63^. By comparison of the activity of the synthesized complexes with similar complexes reported in the literature, it is noted that an enhancement in the activity of the compounds under examination towards the selected species of bacteria^64–66^.

## Conclusions

We have successfully prepared new VO^2+^, Ni^2+^ and Cu^2+^ metal chelates with isatin based ligand; the compounds were characterized using analytical, spectral, thermal techniques. DFT studies were performed to elucidate the structures and relative stability of the ligand tautomers, as well as the coordination geometry of the metal complexes of the most stable form. The compounds were evaluated for their antidiabetic potential, cytotoxic activity and antibacterial efficacy. According to the results obtained, important points can be highlighted:

- The ligand ligates as a neutral or bi-anionic bidentate chelating agent as indicated from FT-IR and mainly exists in keto form.
- Molar conductance of the chelates refers to the non-electrolytic character.
- The electronic spectral data, magnetic moment and DFT calculations of the complexes confirm square-pyramidal geometry for VO^2+^ complex, tetrahedral and distorted square planar geometry for Ni^2+^ and Cu^2+^ complexes, respectively.
- The H_2_L ligand exhibits a relatively small HOMO-LUMO energy gap (3.23 eV), that enhances its softness and reactivity toward metal ion coordination.
- The complexes showed higher electrophilicity (ω) values compared to the ligand, indicating that the studied chelates possess greater biological performance than the free ligand.
- The α-amylase inhibitory activity of ligand enhanced upon complexation with VO^2+^ and Cu^2+^ ions.
- VO^2+^ complex (**1**) possesses strong anticancer performance against hepatic cancer cell (HepG-2) compared with the ligand, Ni^2+^ and Cu^2+^ complexes. The cytotoxic property of the ligand (H_2_L) and its [H_2_LNi(OH)_2_] complex (**2**) against human lung fibroblast normal cell line (WI-38) cells revealed good results.
- Antibacterial activity significantly increases upon coordination. The Cu^2+^ complex (**3**) is the most potent antibacterial agent and possesses the highest inhibition zone towards *S. aureus*,* E. coli* and *K. pneumonia* microorganisms.
- The results suggested that metal complexation improved the biological activity of the ligand.
- The biological features of the VO^2+^, Ni^2+^, and Cu^2+^ complexes and the ligand (H₂L) reported here are promising for future applications; however, the present study remains exploratory. Future work will involve more detailed and systematic investigations, including expanded biological evaluations and deeper structure–activity relationship studies, to further validate and strengthen these findings.

## Supplementary Information

Below is the link to the electronic supplementary material.

Supplementary Material 1

## Data Availability

This published article and its supplemental information file contain all of the data generated or analyzed during this investigation.
