# Supplementary material for: Synthesis, spectroscopic investigation, theoretical insights via DFT and biological assessment of some isatin-based metal complexes
Source: Sci Rep. 2026 Apr 22;16:13151. doi: 10.1038/s41598-026-41979-1 (PMC13103376; doi:10.1038/s41598-026-41979-1)
Supplement: Supplementary file 1 — Supplementary Material 1 [file 41598_2026_41979_MOESM1_ESM.docx]

**Supplementary materials**

**Synthesis, spectroscopic investigation, theoretical insights *via* DFT and biological assessment of some isatin-based metal complexes**

Ohyla A. EL-Gammal^1^[[1]](#footnote-1)^*^, Hanaa A. El-Boraey^2^ Dina A. Tolan^2,3^

***1. Physical methods (Instrumentations)***

The microanalyses (C, H, N) were achieved at Cairo University, Micro Analytical Center, using CHNS-932 (LECO) Vario Elemental Analyzer. Metal and halide ions of metal complexes were determined using the standard methods. The infrared spectra of ligand and its complexes were obtained using Nenexeus-Nicolidite-640-MSA FT-IR spectrophotometer (4000-400 cm^-1^), Thermo-Electronics Co in KBr discs. The ^1^H NMR spectrum was recorded in DMSO-*d_6_* solvent at room temperature using Varian Gemini 200 NMR spectrophotometer at 300 MHz. In addition, the UV-Visible absorption spectra were measured in ethanol solvent using 4802 UV/Visible double beam spectrophotometer. The molar room temperature conductivity of studied complexes was estimated in DMF (10^-3^ M) using a CON 6000 conductivity meter. At room temperature, magnetic susceptibilities of studied complexes were measured by the modified Gouy method using magnetic susceptibility Johnson Matthey balance. The effective magnetic moments were calculated using the formula μ_eff_ = 2.828 (χ_m_T)^1/2^ B.M., where χ_m_ is the molar susceptibility corrected for diamagnetism of all atoms in the compounds. Thermal analyses (TGA/DTG) were carried out by using a Shimadzu DTG/TG-50 thermal Analyzer with heating rate 10°C/ min in nitrogen atmosphere with a flowing rate 20 ml/min in the temperature range 28-800°C using platinum crucibles.

*^[1^Department of Pathology,* *University Hospital, Menoufia University, Shebin El- Kom,32511, Egypt*

*^2^Department of Chemistry, Faculty of Science, Menoufia University, Shebin El- Kom,* *32511, Egypt*

*^3^Department of Chemistry, College of Science and Humanities, Prince Sattam bin Abdulaziz University, Alkharj, 11942, Saudi Arabia]*

*Corresponding author. Tel.: +2- 01098799079; E-mail address: ohyla55@yahoo.com (Ohyla A. El-Gammal).

***2.Biological studies***

***2.1. Antidiabetic activity***

The tested sample was dissolved in minimum amount of 10% DMSO and was further dissolved in buffer ((Na_2_HPO_4_/NaH_2_PO_4_ (0.02 M), NaCl (0.006 M) at pH 6.9) to give concentrations ranging from 7.8 to 1000 μg/mL. A volume of 200 μL of α-amylase solution (2 units/mL) was mixed with 200 μL of the extract and was incubated for 10 min at 30 °C. Thereafter 200 μL of the starch solution (1% in water (w/v)) was added to each tube and incubated for 3 min. The reaction was terminated by the addition of 200 μL DNSA reagent (12 g of sodium potassium tartrate tetrahydrate in 8.0 mL of 2 M NaOH and 20 mL of 96 mM of 3,5-dinitrosalicylic acid solution) and was boiled for 10 min in a water bath at 85–90 °C. The mixture was cooled to ambient temperature and was diluted with 5 mL distilled water and the absorbance was measured at 540 nm using a UV-Visible spectrophotometer. The blank with 100% enzyme activity was prepared by replacing the tested sample with 200 μL of buffer. A blank reaction was similarly prepared using the tested sample at each concentration in the absence of the enzyme solution. A positive control sample was prepared using Acarbose and the reaction was performed similarly to the reaction with tested sample as mentioned above [34,35]. The α-amylase inhibitory activity was expressed as percent inhibition and was calculated using the equation given below: The % α-amylase inhibition was plotted against the tested sample concentration and the IC_50_ values were obtained from the graph.

% α amylase inhibition= [(Abs_control_ − Abs_Sample_) /Abs_Control_] ×100%

***2.2. Cytotoxicity assays***

The cell lines (HepG-2) were obtained from the American Type Culture Collection (ATCC, Rockville, MD) and screened at the regional center for mycology and biotechnology, Al-Azhar university, Cairo, Egypt. For antitumor assays, the tumor cell lines were suspended in medium at concentration 5x10^4^ cell/well in Corning® 96-well tissue culture plates, then incubated for 24 hr. The tested compounds were then added into 96-well plates (three replicates) to achieve eight concentrations for each compound. Six vehicle controls with media or 0.5 % DMSO were run for each 96 well plate as a control. After incubating for 24 hr, the numbers of viable cells were determined by the MTT test. Briefly, the media was removed from the 96 well plate and replaced with 100 µl of fresh culture RPMI 1640 medium without phenol red then 10 µL of the 12 mM MTT stock solution (5 mg of MTT in 1 mL of PBS) to each well including the untreated controls.

The 96 well plates were then incubated at 37°C and 5% CO_2_ for 4 hrs. An 85 µL aliquot of the media was removed from the wells, and 50 µL of DMSO was added to each well and mixed thoroughly with the pipette and incubated at 37°C for 10 min. Then, the optical density was measured at 590 nm with the microplate reader (SunRise, TECAN, Inc, USA) to determine the number of viable cells and the percentage of viability was calculated as [(ODt/ODc)]x100% where ODt is the mean optical density of wells treated with the tested sample and ODc is the mean optical density of untreated cells. The relation between surviving cells and drug concentration is plotted to get the survival curve of each tumor cell line after treatment with the specified compound. The 50% inhibitory concentration (IC_50_), the concentration required to cause toxic effects in 50% of intact cells, was estimated from graphic plots of the dose response curve for each conc. using Graphpad Prism software (San Diego, CA. USA) [36,37].

**Cell line Propagation:**

The cells were grown on RPMI-1640 medium supplemented with 10% inactivated fetal calf serum and 50µg/mL gentamycin. The cells were maintained at 37ºC in a humidified atmosphere with 5% CO_2_ and were subcultured two to three times a week.

***2.3. Antimicrobial assay***

***Method of testing***

The sterilized media was poured onto the sterilized Petri dishes (20-25) mL, each petri dish) and allowed to solidify at room temperature. Microbial suspension was prepared in sterilized saline equivalent to McFarland 0.5 standard solution (1.5x 10^5^ CFU mL^-1^) and its turbidity was adjusted to OD= 0.13 using spectrophotometer at 625 nm. Optimally, within 15 min. after adjusting the turbidity of the inoculum suspension, a sterile cotton swab was dipped into the adjusted suspension and was flooded on the dried agar surface then allowed to dry for 15 min. with lid in place. Wells of 6 mm diameter was made in the solidified media with the help of sterile borer. 100 μL of the solution of the tested compound was added to each well with the help of micropipette. The plates were incubated at 37 °C for 24 h in case of antibacterial activity. Standard discs of Ampicillin (antibacterial agent Gram +ve) and Gentamicin (Gram -ve) were used as positive controls for antibacterial activity but the filter paper disc impregnated in DMSO was used as negative control. The experiment was carried out in triplicate and zones of inhibition were measured in mm scale [38].

***2.4. Statistical analysis***

Differences between samples in the same type of bacteria (or fungi) were analyzed using one way analysis of variance (ANOVA), followed by Duncan multiple comparisons test using SPSS package version “22” for Windows. Values are represented as mean ± S.E. and p < 0.05 was considered statistically significant, p <0.01 was considered highly significant and p <0.001 was considered very highly significant.


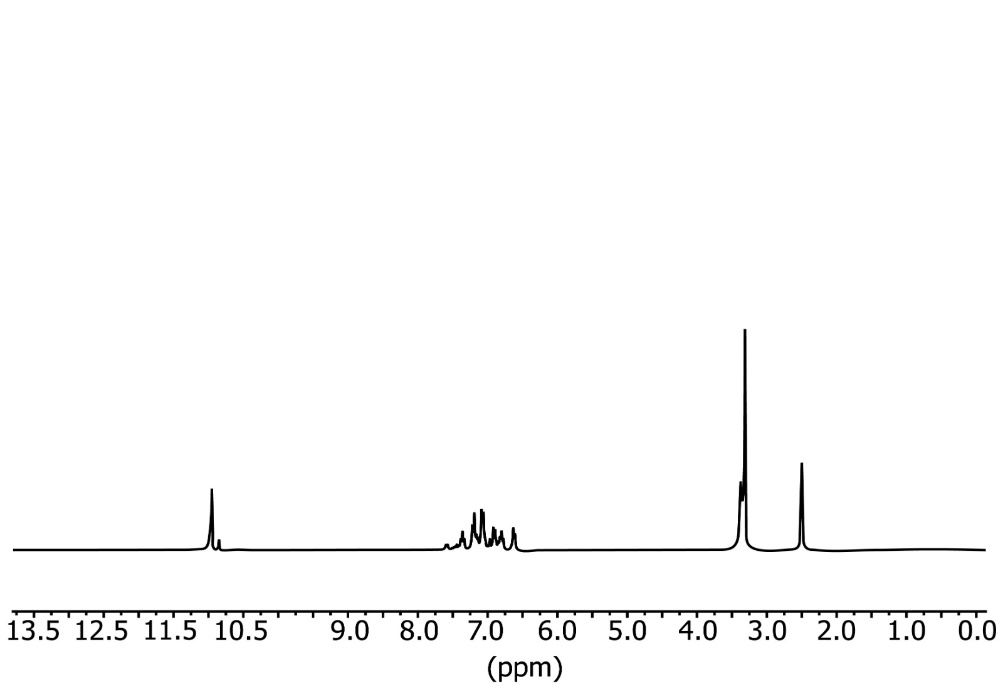


**Fig.1S:** ^1^H NMR spectrum of ligand.


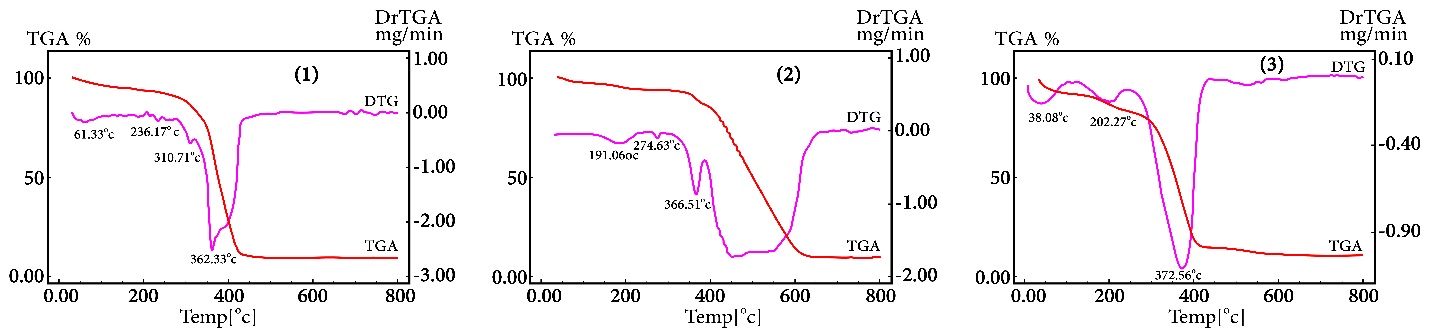


**Fig. 2S:** TG/DTG curves of **(1,2,3)** complexes**.**

**Figure 3S**: The cytotoxic activity of H_2_L and its metal complexes against tested cell line.

**Table 1S:** The Cytotoxic activity of ligand and its complex (**2**) against human lung fibroblast normal cell line (WI-38) cells.

| **Sample conc. (µg/mL)** |  | **Ligand** |  |  | **Complex (2)** |  |
| --- | --- | --- | --- | --- | --- | --- |
|  | **Viability %** | **Inhibitory %** | **S.D. (±)** | **Viability %** | **Inhibitory %** | **S.D. (±)** |
| 500 | 37.42 | 62.58 | 2.42 | 17.18 | 82.82 | 1.81 |
| 250 | 86.75 | 13.25 | 1.62 | 40.81 | 59.19 | 1.46 |
| 125 | 97.41 | 2.59 | 1.20 | 57.57 | 42.43 | 0.84 |
| 62.5 | 99.76 | 0.24 | 0.30 | 72.41 | 27.59 | 1.07 |
| 31.25 | 100 | 0 |  | 84.60 | 15.40 | 1.35 |
| 15.6 | 100 | 0 |  | 93.65 | 6.35 | 0.88 |
| 7.8 | 100 | 0 |  | 99.21 | 0.79 | 0.52 |
| 3.9 | 100 | 0 |  | 100 | 0.00 | 0.00 |
| 0 | 100 | 0 |  | 100 | 0 |  |

**Table 2S: Antimicrobial activity of ligand and its metal chelates (1,2,3).**

| **No.** | **Compound** | **Zone of inhibition (mm)** | | | |
| --- | --- | --- | --- | --- | --- |
|  |  | **Gram positive bacteria** (G+) | | **Gram negative bacteria** (G-) | |
|  |  | *Staphylococcus aureus* *(ATCC:13565)* | *BacillusSubtilis*  *(DSM:1088)* | *Escherichia coli* *(ATCC:10536)* | *Klebsiella pneumonia*  *(ATCC:10031)* |
|  | Ampicillin | 20.7±0.6 | 21.3±0.6 | --- | ---- |
|  | Gentamicin | -- | --- | 27.0±1.0 | 25.3 ±0.6 |
|  | H_2_L | NA | NA | NA | NA |
| **1** | [H_2_LVO(OH)_2_].3H_2_O | 15.3±0.6 | NA | NA | 9.3±0.6 |
| **2** | [H_2_LNi(OH)_2_] | 12.3±0.6 | 13.3±0.6 | 10.3±0.6 | 8.3±0.6 |
| **3** | [LCu(H_2_O)_2_].EtOH | 15.7±0.6 | 12.7±0.6 | 13.0±1.0 | 19.7±0.6 |

- Zone of inhibition is expressed in the form of Mean ± Standard deviation (mm).
- NA: No activity.
- Well diameter (6 mm).
- 100µL was tested.

1. [↑](#footnote-ref-1)
